# Supplementary material for: Volatilomics and Macro-Composition Analyses of Primary Wuyi Rock Teas of Rougui and Shuixian Cultivars from Different Production Areas
Source: Plants (Basel). 2024 Aug 9;13(16):2206. doi: 10.3390/plants13162206 (PMC11359256; doi:10.3390/plants13162206)
Supplement: Supplementary file 1 [file plants-13-02206-s001.zip › Supplementary Figures.pdf]

plants

Supplementary Figures

Volatilomics and Macro-Composition Analyses of Wuyi Rock Primary Teas of Rougui and Shuixian Cultivars from Different Production Areas

Lixuan Zhang [1], Chengzhe Zhou [1], Cheng Zhang [1], Mengcong Zhang [1], and Yuqiong Guo [1,3,4,*]

[1] Anxi College of Tea Science, College of Horticulture, Fujian Agriculture and Forestry University, Fuzhou 350002, China
[2] Tea Green Cultivation and Processing Collaborative Innovation Center, Anxi County, Quanzhou, 362400, China
[3] Tea Industry Research Institute, Fujian Agriculture and Forestry University, Fuzhou 350002, China
* Correspondence: guoyq828@163.com

This PDF file includes: Supplementary Figures S1–S2

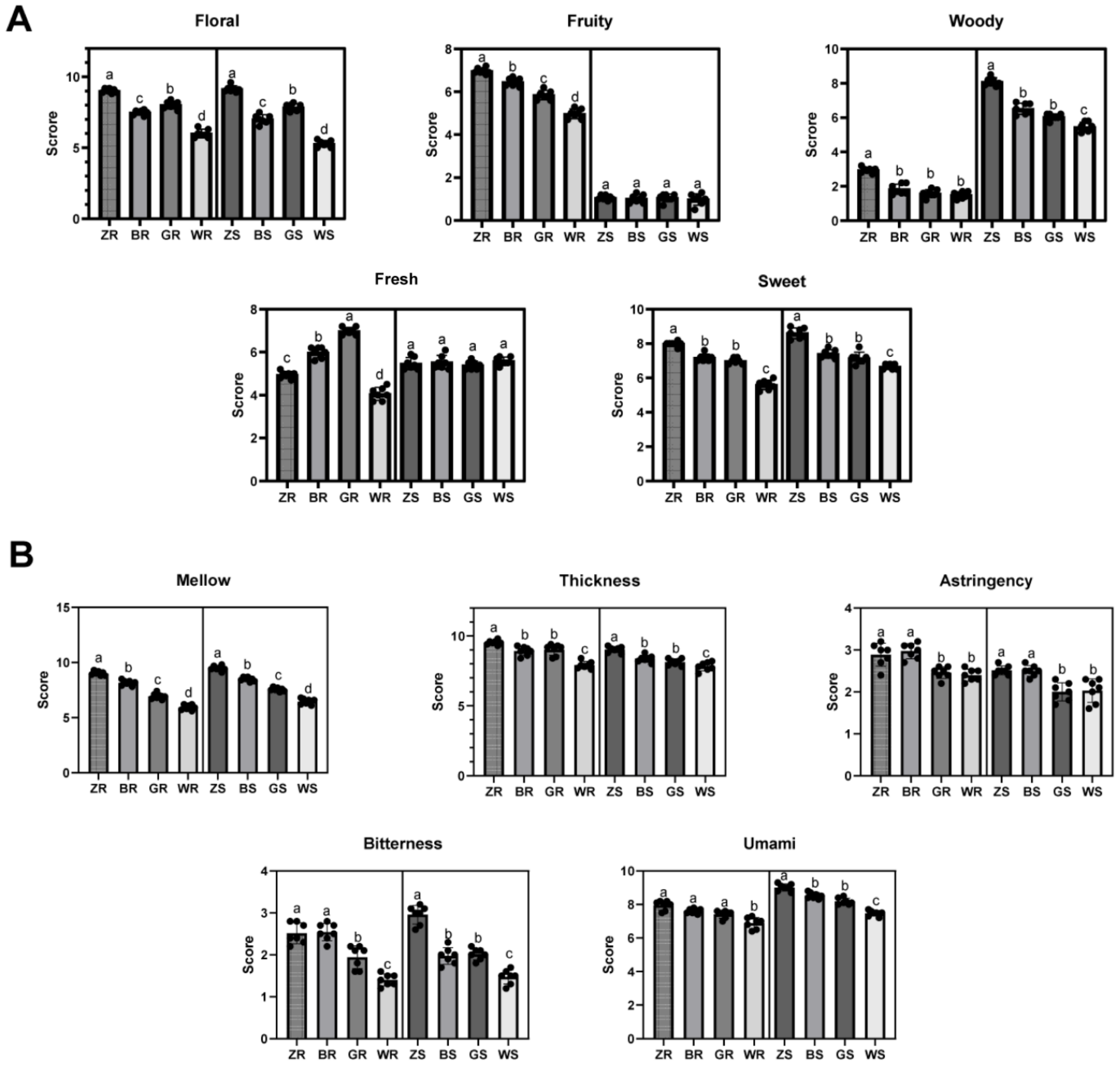

Figure S1. The significant difference analysis of aroma (**A**) and taste (**B**) attributes in 'Rougui' and 'Shuixian' PWRTs from different production areas. The various small letters represent significant differences ($p < 0.05$).

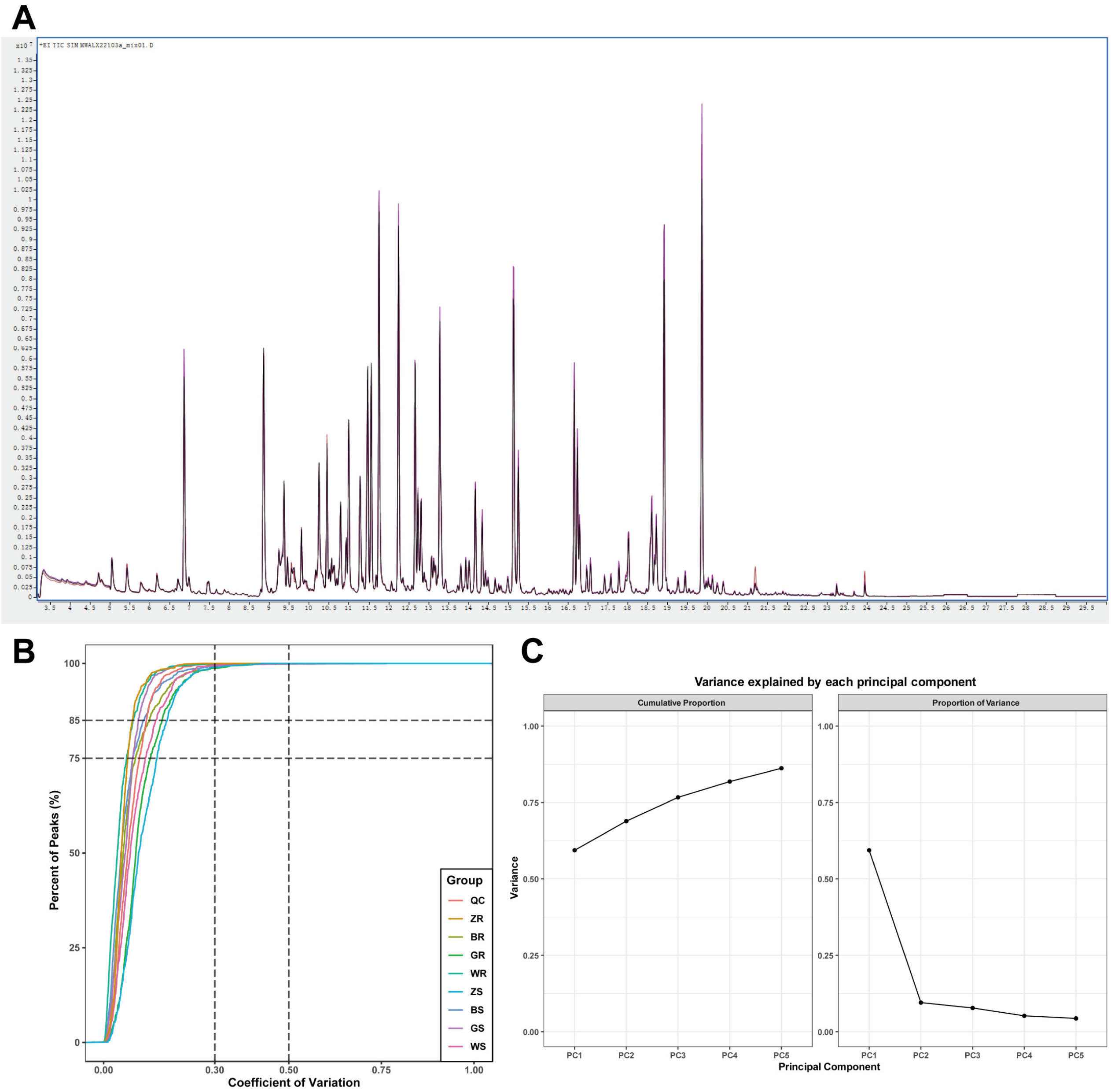

Figure S2. Sample quality control analysis for volatile determination. (**A**) Total ion chromatogram of mass spectrometry analysis of the mixed sample for quality control. (**B**) Coefficient of variation analysis. (**C**) The proportion of variance for the first five principal components from PCA model.
